# Non-invasive myocardial work as an independent predictor of postprocedural NT-proBNP in elderly patients undergoing transcatheter aortic valve replacement

**DOI:** 10.1007/s11357-024-01302-0

**Published:** 2024-08-08

**Authors:** Zsuzsanna Ladányi, Tímea Bálint, Alexandra Fábián, Adrienn Ujvári, Tímea Katalin Turschl, Dávid Nagy, Éva Straub, Csaba Fejér, Endre Zima, Astrid Apor, Anikó Ilona Nagy, Tímea Szigethi, Roland Papp, Levente Molnár, Attila Kovács, Mihály Ruppert, Bálint Károly Lakatos, Béla Merkely

**Affiliations:** Heart and Vascular Center, Semmelweis University, Budapest, Hungary

**Keywords:** Echocardiography, Myocardial work, Valvular disease, Aortic stenosis, Transcatheter aortic valve replacement, N-terminal pro-beta natriuretic peptide

## Abstract

Aortic stenosis has become the most prevalent valvular disease with increasing life expectancy and the ageing of the population, representing a significant clinical burden for health care providers. Its treatment has been revolutionized by transcatheter aortic valve replacement (TAVR) as a safe and minimally invasive option for elderly patients. Left ventricular (LV) functional measurement is of particular importance before TAVR, however, increased afterload significantly influences the conventional echocardiographic parameters. Non-invasive myocardial work examines myocardial deformation in the context of instantaneous LV pressure, thus, it might be a more reliable measure of LV function. Accordingly, we aimed to study non-invasive myocardial work and its relationship with functional outcome following TAVR.

We enrolled 90 TAVR candidates (80 [75–84] years; 44% female). Using echocardiography, we quantified ejection fraction (EF), global longitudinal strain (GLS), global myocardial work index (GWI) and global constructive work (GCW) before and 12 months after the procedure. Serum NT-proBNP levels were also measured. EF did not change (52.6 ± 13.1 vs. 54.2 ± 10.5%; *p* = 0.199), while GLS increased (-13.5 ± 4.6 vs. -15.2 ± 3.8%; *p* < 0.001). GWI decreased (1913 ± 799 vs. 1654 ± 613 mmHg%; *p* < 0.001) and so did GCW (2365 ± 851 vs. 2177 ± 652 mmHg%; *p* = 0.018). History of atrial fibrillation (AF) (β = 0.349) and preprocedural GCW (β = -0.238) were independent predictors of postprocedural NT-proBNP (*p* < 0.001).

GLS, GWI and GCW changed after TAVR while there was no alteration in EF. The preprocedural GCW and history of AF were independent predictors of postprocedural NT-proBNP. Accordingly, myocardial work indices may help patient selection and the prediction of the functional outcome in this population.

**Supplementary Information:**

The online version contains supplementary material available at 10.1007/s11357-024-01302-0.

## Background

With increased life expectancy and aging of the population, aortic valve stenosis (AS) has become increasingly common. Degenerative aortic valve disease affects over 25% of all patients over the age of 65. Most patients have only mild thickening and normal valve function, called aortic sclerosis. However, 2%–5% of these patients have significant AS [1, 2]. It is now the most common valvular disease in developed countries representing a significant burden for health care providers [3, 4]. As this is primarily the disease of older adults, it is often associated with comorbidities that complicate treatment. AS affects millions worldwide, with a substantial portion of elderly patients requiring intervention. Traditionally, surgical aortic valve replacement (SAVR) was the primary treatment, but transcatheter aortic valve replacement (TAVR) has revolutionized care by providing a minimally invasive option. TAVR is now a standard treatment for elderly patients with high surgical risk [5–7], and is increasingly recommended for younger patients with lower operative risk [8, 9].

As the population ages, the incidence of AS and the need for effective, less invasive treatments like TAVR will continue to rise. Tailored patient selection and risk stratification is of the utmost importance in such a complex and fragile population. Some studies even suggest that the symptom-guided management decisions in AS may need revision [10]. A key element of preprocedural patient assessment before TAVR is left ventricular (LV) function, most commonly by echocardiography. Ejection fraction (EF) is the mainstay parameter of LV function, also used as a measure to discriminate AS of different hemodynamic profiles (e.g. normal flow-high gradient, low flow-low gradient AS etc.). Notably, speckle-tracking echocardiography-derived global longitudinal strain (GLS) has been embraced as a more sensitive measure of LV systolic function in the last decade, with established superior prognostic value [11].

However, despite the wide acclaim and utilization of these parameters, their load-dependency should not be neglected. Therefore, it is necessary to explore new diagnostic and predictive measures to improve patient outcomes and optimize treatment strategies. To mitigate the limitations of conventional parameters of LV systolic function, the concept of myocardial work (calculated by adjusting myocardial deformation to the instantaneous LV pressure) has been recently proposed and tested in various clinical scenarios [12], suggesting that myocardial work indices might be robust markers of patient outcomes even in diseases with severely altered loading conditions, such as AS. Accordingly, several studies have investigated their prognostic significance. For example, both in heart failure and myocardial infarction patients, impaired myocardial work has been associated with worse clinical outcomes, including higher rates of hospitalization, cardiovascular events, and mortality [13, 14].

Apart from echocardiographic parameters, a number of other exams are also valuable during the follow-up of AS patients, including laboratory tests. N-terminal prohormone of brain natriuretic peptide (NT-proBNP) is a biomarker indicative of cardiac stress and heart failure [15]. Elevated levels of NT-proBNP are associated with poor cardiac function and are predictive of adverse cardiovascular events [16]. This marker is widely used in clinical practice to assess the severity of heart failure and to monitor treatment outcomes. Additionally, in patients with AS the increase of NT-proBNP levels is also associated with adverse outcomes [17–19]. Furthermore, TAVR patients with increased NT-proBNP had an increased risk for 5-year and 2-year all-cause and cardiac death in previous studies [20, 21], so this biomarker is a relevant measure for evaluating the effectiveness of the procedure and patients’ outcomes afterwards.

Therefore, we sought to evaluate the myocardial work indices of severe AS patients undergoing TAVR, and to explore the predictive value of preoperative myocardial work regarding the postoperative status of the patients, with the postoperative NT-proBNP levels serving as the endpoint.

## Methods

### Study population

**Fig. 1:**
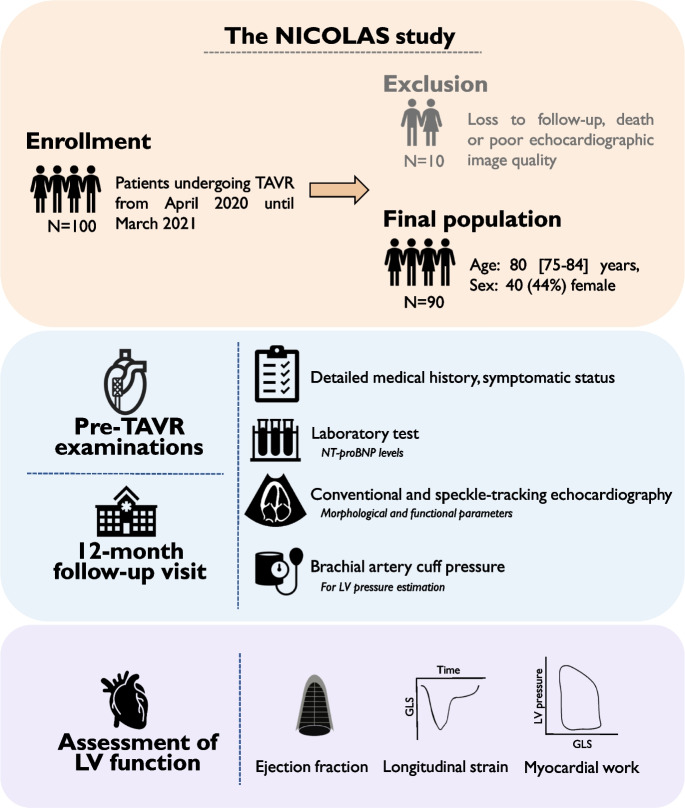
Study design A schematic overview of the study. 100 patients were enrolled, and after the exclusion the final population consisted of 90 people. Prior to the transcatheter aortic valve replacement and at a 12-month follow up visit detailed medical history, and laboratory tests were obtained. The patients underwent conventional and speckle-tracking echocardiographic examinations, along with brachial artery pressure measurement as per protocol. Left ventricular function was quantified at both time points, using ejection fraction, global longitudinal strain and myocardial work indices, to assess their change and association with the clinical characteristics of the patients

In the NICOLAS (*“Non-Invasive measurement of myocardial COntractiLity in patients with Aortic Stenosis”*) study, we prospectively enrolled patients undergoing TAVR in our Institute. In the current analysis (Fig. 1), patients from April 2020 until March 2021 (*n* = 100) were asked to participate in a 12-month follow-up visit after the procedure. The study protocol complies with the Declaration of Helsinki, and participants gave written informed consent to every procedure. The study protocol was approved by the Institutional Ethical Committee (SE-TUKEB No. 2020/115) Detailed medical history, symptomatic status and as a marker of congestion, NT-proBNP levels were obtained both before the procedure and during a 12-month follow-up visit. Echocardiographic exams were performed less than 24 h prior to TAVR, and during a 12-month follow-up visit. The final analysis did not contain patients who were lost to follow-up (mainly due to COVID-19 regulations) (4 patients), died during the 12 months (2 patients) and had poor echocardiographic image quality (the endocardial border was difficult to visualize, thus preventing strain analysis) on either of the exams (4 patients). Accordingly, the final study population consisted of 90 patients.

### Transcatheter aortic valve replacement

TAVR procedure was performed under conscious sedation and local anesthesia in all patients. Transfemoral approach was used in all patients. Self-expandable valves were implanted such as CoreValve/EvolutR/EvolutPro (Medtronic, Minneapolis, MN, USA) (*n* = 40, 44%), Portico (Abbott, Abbott Park, IL, USA) (*n* = 35, 39%) or Acurate Neo/Acurate Neo 2 (Boston Scientific, Marlborough, MA, USA) (*n* = 15, 17%). Computed tomographic (CT) angiography and cardiac CT was used to evaluate arterial pathologies and for valve sizing. The sizes of the implanted valves were 28.8 ± 3.4 mm.

### Conventional echocardiography

Conventional echocardiographic exams were performed using a GE Vivid E95 (equipped with a 4Vc-D matrix-array transducer; GE Healthcare, Horten, Norway) ultrasound system. A standard acquisition protocol consisting of loops from parasternal, apical, and subxiphoid views was used according to current guidelines [22]. LV wall thicknesses and diameters were evaluated in parasternal long-axis view. Relative wall thickness (RWT) was calculated as end-diastolic posterior wall thickness (PWd) multiplied by 2 and divided by LV end-diastolic internal diameter. End-diastolic (LV EDVi) and the end-systolic (LV ESVi) volume indices were quantified using the biplane Simpson method, by indexing the volumes to the body surface area (BSA), and using these data, LV EF was also calculated. Left ventricular outflow tract (LVOT) diameter was measured in parasternal long-axis view, and using pulsed wave Doppler interrogation at its level in apical three-chamber view, LVOT velocity–time integral (VTI) was also calculated. The aortic valve area (AVA) was calculated according to the continuity equation. Pulsed wave Doppler interrogation at the level of the mitral valve coaptation was obtained to determine early (E) and late diastolic (A) peak LV inflow velocities and their ratio (E/A) and deceleration time (DT). Systolic (s′), early (e′), and late diastolic (a′) velocities at the mitral lateral and medial annuli were measured using pulsed-wave tissue Doppler imaging. LV filling pressure was estimated by dividing the transmitral E wave with the tissue Doppler imaging-derived averaged lateral and medial annular e’. M-mode-derived tricuspid annular plane systolic excursion (TAPSE) was calculated as the maximum longitudinal displacement of the tricuspid annulus. Peak pulmonary artery systolic pressure (PASP) was calculated from the tricuspid regurgitant jet signal velocity and right atrial pressure, which was estimated using inferior vena cava diameter and collapsibility. Atrial volumes were estimated using the Simpson method. The left atrial volume index (LAVi) and right atrial volume index (RAVi) were normalized to the BSA.

According to the proposed classification of cardiac involvement in AS [23], the patients were classified as AS Stage 0 (no cardiac damage), Stage 1 (left ventricular damage), Stage 2 (mitral valve and/or left atrial damage), Stage 3 (tricuspid valve and/or pulmonary artery vasculature damage) or Stage 4 (right ventricular damage).

### Speckle-tracking and non-invasive myocardial work analysis

At the end of the echocardiographic exam (after approximately 10 min lying in rest) brachial artery cuff pressure was measured for each subject in the left lateral decubitus position. Speckle-tracking and myocardial work analysis were performed using the dedicated module of a commercially available software solution (EchoPAC v204, GE Healthcare, Chicago, IL, USA). Firstly, the software automatically identified apical four-chamber, two-chamber, and three-chamber views and applied the corresponding region of interest, which could be manually corrected if necessary. Only recordings with a minimum frame rate of 50 FPS were used. Then, segmental and global longitudinal strain values were assessed by speckle-tracking. Acceptance or rejection of a particular segment was guided by the software’s recommendation. In each patient, no more than 2 segments were excluded due to inadequate tracking quality (based on the segment’s time-strain curve characteristics and/or visual assessment). Left ventricular mass index (LVMi) was calculated by indexing the left ventricular mass values (estimated according to the Devereux formula) to the to BSA. After the speckle-tracking analysis, event timings (aortic valve closure, mitral valve opening, mitral valve closure, aortic valve opening) were manually adjusted based on visual assessment of the valves in the apical long-axis view recording. After providing the sum of the systolic brachial artery cuff pressure value and the mean transaortic gradient, and the diastolic brachial artery cuff pressure value to the software, the LV pressure curve was automatically estimated, this method was used during both the pre- and postoperative analysis, as well [24–26]. Therefore, myocardial deformation (i.e., LV GLS) could be evaluated in relation to the estimated instantaneous LV pressure to enable the non-invasive assessment of myocardial work indices: global myocardial work index (LV GWI) is the amount of myocardial work performed by the left ventricle during systole [the integral of LV power (strain rate multiplied by the concomitant LV pressure) and the time between the closure and opening of the mitral valve], global constructive work (LV GCW) can be defined as the work contributing to the pump function (the shortening of myocytes during systole and their elongation during isovolumetric relaxation), global wasted work (LV GWW) is the work not contributing to the pump function (the elongation of myocytes during systole and their shortening during isovolumetric relaxation), and global work efficiency (LV GWE) can be calculated as LV GCW divided by the sum of LV GCW and LV GWW.

### Statistical analysis

Statistical analysis was performed using STATISTICA version 13.4 (TIBCO Software Inc, Palo Alto, CA, USA) and GraphPad Prism 8.0.1 version (GraphPad Software Inc, San Diegom CA, USA). The normal distribution of our variables was verified using the Shapiro–Wilk test. Data are presented as mean ± standard deviation, median (interquartile ranges), or number of patients (percentage), as appropriate. For continuous variables preoperative and follow-up data were compared with the paired Student’s t-test or Wilcoxon signed-rank test; and for categorical variables the chi-square or Fisher’s exact test according to normality. Correlation analysis was performed using Pearson’s or Spearman’s correlation coefficient, according to normality. The independent predictors of various parameters were assessed by performing multivariate regression analysis. P-values < 0.05 were considered statistically significant.

Intra- and interobserver variability of the most relevant parameters were also assessed. The operator of the first measurements (B.K.L.) and a second expert reader (Z.L.) repeated the measurements on a randomly chosen subset of 5–5 preoperative and follow-up echocardiographic exams. Lin's concordance correlation coefficient and coefficient of variation were calculated.

## Results

### Patient characteristics

The baseline characteristics of the patients are presented in Table 1.

**Table 1:** Baseline characteristics of the TAVR patients

Age (years)	80 [75–84]
Female (n)	40 (44%)
Height (cm)	165 [160–173]
Weight (kg)	78 [70–86]
BSA (m^2^)	1.88 [1.75–2.01]
NYHA Stage 2 +	71 (79%)
log EuroScore	11.5 [7.2–18.1]
Angina (n)	30 (33%)
Syncope (n)	12 (13%)
Dyspnea (n)	73 (81%)
Hypertension (n)	87 (97%)
Diabetes mellitus (n)	34 (38%)
Atrial fibrillation (n)	32 (36%)
Ischemic heart disease (n)	43 (48%)
Previous revascularization (n)	29 (32%)
Peripheral artery disease (n)	20 (22%)
Chronic kidney disease (n)	38 (42%)
CIED (n)	18 (20%)
COPD (n)	20 (22%)
Stroke (n)	9 (10%)
Hypothyreosis (n)	8 (9%)
AS stage 0 (n)	4 (4%)
AS stage 1 (n)	18 (20%)
AS stage 2 (n)	55 (61%)
AS stage 3 (n)	3 (3%)
AS stage 4 (n)	10 (11%)
LFLG AS (n)	19 (21%)
Mild postoperative PVL (n)	16 (18%)
Moderate postoperative PVL (n)	11 (12%)

Data are presented as median [interquartile range] or number of patients (%)

*Abbreviations*: *TAVR*  transcatheter aortic valve replacement, *BSA* body surface area, *NYHA* New York Heart Association, *CIED* cardiac implantable electronic device, *COPD* Chronic obstructive lung disease, *AS* aortic valve stenosis, *LFLG* low-flow low-gradient, *PVL* paravalvular leak

The population was 80 [75–84] years of age, and 40 (44%) of them were female. Every patient had symptomatic severe AS, along with numerous other comorbidities. Prior to the TAVR most of the patients (*n* = 71, 79%) were NYHA Stage 2 + and they had a median of 11.5 [7.2–18.1] logistic EuroScore value.

According to the extent of AS-related cardiovascular damage on echocardiography [23], our study population had patients of all stages in terms of cardiac involvement, albeit, presenting most commonly with Stage 2 (*n* = 55, 61%).

### Conventional echocardiographic parameters

The results of the echocardiographic and laboratory examinations are presented in Table 2.

**Table 2:** Echocardiographic and laboratory parameters prior to the TAVR procedure and at the follow-up

	Preoperative	Follow-up visit	p-value
IVSd (mm)	13.4 ± 3.3	11.4 ± 2.1	** < 0.001**
PWd (mm)	12.6 ± 2.7	11.7 ± 2.6	**0.014**
RWT	0.56 ± 0.18	0.53 ± 0.14	0.069
LVMi (g/m^2^)	124.91 ± 37.34	104.81 ± 31.81	** < 0.001**
LV EDVi (ml/m^2^)	60.1 ± 21.9	56.4 ± 19.5	**0.018**
LV ESVi (ml/m^2^)	30.5 ± 18.7	27.1 ± 15.8	**0.013**
TAPSE (mm)	21.9 ± 5.2	21.3 ± 5.7	0.363
TI grade 2 + (n)	9 (10)	14 (16)	0.264
PASP (mmHg)	40.2 ± 13.6	38.4 ± 12.4	0.208
LVOT VTI (cm/s)	21.98 ± 4.87	23.1 ± 6.6	0.080
AI grade 2 + (n)	7 (8)	5 (6)	0.550
AV peak gr. (mmHg)	68.3 ± 28.1	14.5 ± 5.7	** < 0.001**
AV mean gr. (mmHg)	45.6 ± 19.9	7.9 ± 3.0	** < 0.001**
AVA (mm^2^)	70.9 ± 25.1	200.8 ± 59.0	** < 0.001**
MI grade 2 + (n)	12 (13)	10 (11)	0.649
Transmitral E wave (cm/s)	107.6 ± 30.8	107.9 ± 29.5	0.739
Transmitral A wave (cm/s)	102.3 ± 31.3	111.9 ± 43.2	**0.041**
E/A	1.09 ± 0.54	0.94 ± 0.44	0.108
DT (ms)	190.6 ± 64.4	221.8 ± 72.9	** < 0.001**
Mitral lateral s (cm/s)	7.1 ± 2.0	8.5 ± 6.2	**0.032**
Mitral lateral e’ (cm/s)	8.6 ± 5.5	9.0 ± 3.6	0.470
Mitral lateral a’ (cm/s)	9.3 ± 3.8	10.6 ± 3.3	**0.003**
Mitral medial s (cm/s)	5.6 ± 1.7	6.3 ± 1.8	** < 0.001**
Mitral medial e’ (cm/s)	6.3 ± 2.2	7.1 ± 5.2	0.177
Mitral medial a’ (cm/s)	7.7 ± 2.3	8.2 ± 2.4	0.173
E/e’ average	16.36 ± 7.61	14.78 ± 6.84	0.082
LAVi (ml/m^2^)	45.1 ± 17.2	47.2 ± 17.4	0.086
RAVi (ml/m^2^)	32.6 ± 14.0	37.0 ± 14.1	**0.001**
SBP (mmHg)	126 ± 20	137 ± 22	** < 0.001**
DBP (mmHg)	64 ± 12	68 ± 13	0.072
LV EF (%)	52.6 ± 13.1	54.2 ± 10.5	0.199
LV GLS (%)	-13.5 ± 4.6	-15.2 ± 3.8	** < 0.001**
LV GWI (mmHg%)	1913 ± 799	1654 ± 613	** < 0.001**
LV GCW (mmHg%)	2365 ± 851	2177 ± 652	**0.018**
LV GWW (mmHg%)	260 ± 158	294 ± 144	0.065
LV GWE (%)	88.2 ± 7.1	86.7 ± 7.3	**0.041**
NT-proBNP (pg/mL)	1254[604–3037]	627[364–1435]	** < 0.001**

Data are presented as mean ± SD or number of patients (%)

Values with a significant difference are presented in bold

*Abbreviations*: *TAVR* transcatheter aortic valve replacement, *IVSd* interventricular septal thickness, *PWd* posterior wall thickness, *RWT* relative wall thickness, *LVMi* left ventricular mass index, *LV* left ventricular, *EDVi* end-diastolic volume index, *ESVi* end-systolic volume index, *TAPSE* tricuspid annular plane systolic excursion, *TI* tricuspid valve insufficiency, *PASP* pulmonary artery systolic pressure, *LVOT* left ventricular outflow tract, *VTI* velocity time integral *AI* aortic valve insufficiency, *AV* aortic valve, *AVA* aortic valve area, *MI* mitral valve insufficiency, *DT* deceleration time, *LAVi* left atrial volume index, *RAVi* right atrial volume index, *SBP*  systolic blood pressure, *DBP* diastolic blood pressure, *EF* ejection fraction, *GLS* global longitudinal strain, *GWI*  global myocardial work index, *GCW*  global constructive work, *GWW*  global wasted work, *GWE*  global myocardial work efficiency, *NT-proBNPT* N-terminal prohormone of brain natriuretic peptide

Both LV EDVi and LV ESVi decreased significantly by the time of the follow-up visit, and so did the interventricular septal thickness (IVSd) and PWd, however, the RWT did not change. LVMi also decreased significantly following the TAVR. In accordance with the procedure, the aortic gradients markedly decreased, while the AVA increased. The transmitral A wave became significantly larger, as well as the DT, while E/A did not change. We also found an increase in the mitral lateral s, a’ and the mitral medial s, however, E/e’ remained unchanged. Regarding the right ventricle, neither TAPSE, nor PASP showed any change during the follow-up. LAVi did not change by the time of the follow-up, however, RAVi increased significantly. Notably, the systolic blood pressure of the patients was higher during the follow-up visit than prior to the TAVR.

### Left ventricular systolic function

**Fig. 2:**
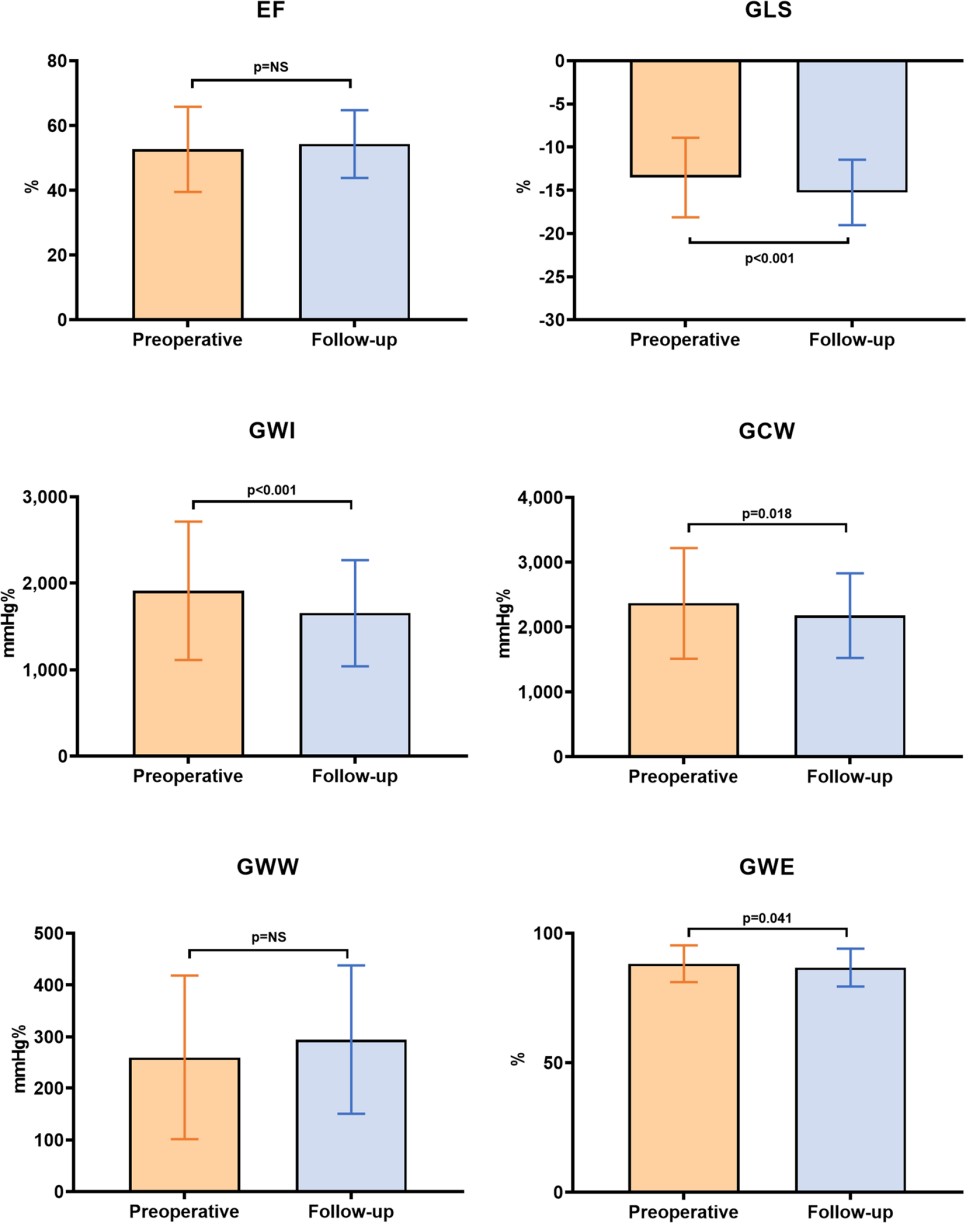
Functional echocardiographic measures of the patients prior to TAVR and during the follow-up visit. EF and GWW did not change, the absolute value of GLS increased significantly, while GWI, GCW and GWE all decreased significantly. EF = ejection fraction, GLS = global longitudinal strain, GWI = global myocardial work index, GCW = global constructive work index, GWW = global wasted work, GWE = global myocardial work efficiency, NS = non-significant

The parameters of LV systolic function are also included in Table 2. LV EF remained unchanged by the time of the 12-month follow-up visit. However, the absolute value of LV GLS increased significantly, while LV GWI, LV GCW and LV GWE all decreased by this time (Fig. 2). Despite these results of the whole population, a considerable proportion of the cohort (*n* = 27, 30%) showed increasing GWI values (Supplementary Table 1). These patients had larger preoperative LV volumes, and higher E/A and E/e’ values than those patients, whose GWI did not increase. Their preoperative TAPSE, LV EF, LV GLS, GWI, GCW and GWE values were also significantly worse.

### The relationship of myocardial work indices and the clinical data

By the time of the follow-up visit, the NT-proBNP levels of the patients improved significantly (1254 [604–3037] vs. 627 [364–1435] pmol/L; *p*< 0.001). Using multivariate regression analysis, we determined the independent preoperative predictors of the postoperative NT-proBNP levels, our models are presented as Tables 3 and 4. The correlation analysis for the parameters used in the models are presented in Supplementary Table 2. In the first model, which included preoperative LV GLS as a measure of systolic function, only a history of preoperative atrial fibrillation proved to be an independent predictor. In the second model, by replacing LV GLS with LV GCW as a parameter of systolic function, along with atrial fibrillation it was also an independent predictor of the postoperative NT-proBNP, improving the strength of the model.

**Table 3:** Independent preoperative predictors of postoperative NT-proBNP levels – Model 1

Preoperative parameters	β	p-value	Std. Err	R^2^	Cumulative p-value
Age	0.106	0.338	908	0.300	**0.001**
Female sex	-0.016	0.871			
Syncope	0.134	0.182			
Angina	-0.132	0.213			
Dyspnea	-0.076	0.440			
AS stage	0.129	0.279			
Diabetes mellitus	0.144	0.165			
Atrial fibrillation	**0.370**	** < 0.001**			
LV GLS (%)	0.206	0.095			

Values with a significant predictive value are presented in bold

*Abbreviations*: *NT-proBNP* N-terminal prohormone of brain natriuretic peptide, *AS* aortic stenosis, *LV GLS* left ventricular global longitudinal strain

**Table 4:** Independent preoperative predictors of postoperative NT-proBNP levels – Model 2

Preoperative parameters	β	p-value	Std. Err	R^2^	Cumulative p-value
Age	0.079	0.456	901	0.311	** < 0.001**
Female sex	0.007	0.948			
Syncope	0.141	0.157			
Angina	-0.129	0.218			
Dyspnea	-0.085	0.390			
AS stage	0.123	0.288			
Diabetes mellitus	0.137	0.218			
Atrial fibrillation	**0.349**	**0.001**			
LV GCW (mmHg%)	**-0.238**	**0.046**			

Values with a significant predictive value are presented in bold

*Abbreviations*: *NT-proBNP*  N-terminal prohormone of brain natriuretic peptide, *AS* aortic stenosis, *LV GCW* left ventricular global constructive work

We also investigated the predictors of postoperative myocardial work indices in Supplementary Tables 3 and 4. The correlation analysis for the parameters used in the models of Supplementary Tables 3 and 4 are presented in Supplementary Table 5. Female sex, AS stage, the presence of diabetes mellitus proved to be independent predictors of both postoperative LV GWI and GCW, however, regarding LV GWI, stroke in the medical history and the preoperative length of the QRS also appeared as independent predictors.

27 (30%) patients had mild (*n* = 16; 18%) or moderate (*n* = 11; 12%) postoperative paravalvular leak (PVL). Using ANOVA, we checked if there was a difference in NT-proBNP levels or myocardial work values between the no PVL, mild PVL and moderate PVL groups, and found that the NT-proBNP levels did indeed differ between the groups (*p*  = 0.033), while the myocardial work values were comparable (GWI *p*  = 0.221; GCW *p*  = 0.327; GWW *p*  = 0.202; GWE *p*  = 0.480).

19 patients (21%) had low-flow low-gradient (LFLG) AS. When comparing them to the high gradient AS patietns, we found lower GWI, GCW and GWE values in the LFLG population during the preoperative exam, and the LFLG patients showed no change in myocardial work values by the time of the follow-up visit, while the high flow AS patients had decreasing GWI and GCW values (Supplementary Table 6).

### Inter- and intraobserver variability

The intra- and interobserver variability analysis of LV GLS and the key myocardial work indices demonstrated good agreement in the assessment of these measures (Supplementary Table 7).

## Discussion

In the present study, we investigated the changes of LV functional measures, most notably myocardial work index parameters following TAVR. The most important findings can be summarized as follows: (i) GWI, GCW and GWE significantly decreases following the valve replacement; (ii) the decrease in GWI is not uniform, as a considerable proportion of the cohort displays increases in GWI; (iii) several clinical factors independently determine the postoperative value of GWI and GCW; (iv) GCW is an independent predictor of postoperative NT-proBNP levels, while conventional measures of LV function are not.

A unique feature of transcatheter valve interventions is the prompt significant hemodynamic effect. While long-standing pressure overload is associated with characteristic alterations of the chamber, LV afterload immediately decreases after the procedure, leading to complex morphological and functional changes. This LV reverse remodeling is a solid indicator of improving hemodynamics after TAVR with established prognostic role [27], and our findings of decreasing LV volumes and wall thicknesses, as well as reduced LVMi, are in unison with the literature discussing the effects of TAVR [28]. Regarding the LV systolic function, EF did not change after TAVR. Notably, EF is heavily dependent on both preload and afterload [29], therefore, it may not be an informative marker to assess the actual changes in LV function following the procedure. A subtle systolic dysfunction prior to TAVR is suggested by the pathologically reduced LV GLS values of the cohort [30]. Similarly to our results, postprocedural increase in the reduced LV GLS values is a common finding after TAVR [31–34]. Nevertheless, GLS is also significantly influenced by multiple factors, such as loading conditions, heart rate and LV geometry [11]. Our research group previously demonstrated that GLS reflects the connection of cardiac contractility to afterload (termed ventriculoarterial coupling) rather than mere contractility in rat models of hemodynamic overload-induced heart failure [35]. Based on these findings, the preprocedural GLS values inherently carry the substantial influence of LV afterload which also limit their application in this population as sensitive markers of the LV contractile state.

The concept of myocardial work estimation may overcome the significant limitation of the load-dependent nature of LV deformation [36]. Previous studies have already shown the added clinical value of myocardial work in various clinical conditions, such as heart failure with reduced and with preserved ejection fraction, or ischemic heart disease [13, 14]. Of note, the strength of this kind of afterload-adjusted deformation analysis may be the most evident in cardiac diseases in which the primary drive of the disease is the derangements of LV hemodynamic load. Recently, GWI was also proven to be a predictor of adverse outcome in patients undergoing TAVR outperforming EF and GLS in this regard [37]. In rat models of pressure- or volume-overload induced heart failure and the athlete’s heart, GWI and GCW strongly correlated with load-independent invasive measures of LV contractility [38, 39]. Based on these experimental data, myocardial work may be implemented as a non-invasive marker of LV inotropy in the clinical practice.

Our results of decreasing postprocedural LV GWI, GCW and GWE are in accordance with the findings of Franco et al. [40] and extend the results of De Rosa et al., who found decreasing GWI and GCW values two weeks after TAVR [41]. Importantly though, this change was not consistent in the entire cohort as 27 patients had increasing GWI values. The reason behind this might be found in the pathophysiological development of AS: as most of our patients still had compensated LV function, the pressure overload was counterbalanced by increased LV contractility, which may be reflected by high myocardial work values; therefore, when the pathologically high afterload was resolved by the TAVR, the need for the compensatory contractility augmentation vanished, leading to decreased myocardial work accordingly [42]. On the other hand, in those patients, whose LV inotropic state started to deteriorate by the valvular dysfunction, the significant afterload reduction by TAVR potentially promoted normalization of LV contractility with consequential increase in myocardial work values [23, 43]. Our findings of lower preoperative LV functional parameters in the GWI-increase group support this theory and it is also underpinned by the strong independent predictive role of preprocedural AS stage in the postoperative GWI and GCW values. In that sense, the TAVR-related changes of myocardial work can be only interpreted in the context of the concomitant cardiac remodeling [44]. When comparing LFLG and normal flow AS patients we found lower myocardial work values in the LFLG population before the TAVR, which can be explained by their low-flow state. Interestingly, by the time of the follow-up visit the LFLG patients showed no improvement regarding myocardial work, while the high flow AS group had decreasing GWI and GCW values, which is similar to the findings of de Rosa et al. [41], and could be an important aspect to consider during patient selection.

Notably, we found an increase in systolic blood pressure after the procedure, which has already been observed in other studies [45]. Importantly, its effect on myocardial work indices is not negligible, and should be taken into consideration when interpreting the work values.

We found that the myocardial work indices during the follow-up visit were associated with a number of clinical characteristics of the patients. Sex has been linked to GWI, GCW and GWW values in previous studies [46]. Notably, in an abdominal aortic banded rat model of pressure-overload induced left ventricular hypertrophy, the reversibility of fibrosis and pathological remodeling after debanding the animals was noted to be higher in females [47]. Moreover, several studies have focused their attention on the growing evidence that women benefit more in terms of outcomes after TAVR when compared to men, especially in the long-term [48]. Diabetes mellitus being a predictor of lower myocardial work indices during the follow-up can also be anticipated, considering that the disease leads to microvascular injury [49], which may limit the degree of reverse remodeling after TAVR [50]. The predictive role of the preoperative length of the QRS may be associated with LV dyssychrony, which has been suggested to be strongly afterload-dependent, which is also reflected in the myocardial work values [51, 52]; moreover, previous studies found left bundle branch block to be associated with decreased GWI and GWE, and with increased GWW, however, GCW remained unchanged in left bundle branch block patients [53]. Interestingly, we found that stroke events prior to TAVR were also predictors of GWI at the follow-up visit. Stroke is a dreaded perioperative complication of TAVR [51, 54], however, its association with myocardial work is yet to be studied. Subtle LV dysfunction shown by lower GLS values was previously reported in stroke patients [55]. Our findings suggest prolonged dysfunction after the events and changes in not only GLS but myocardial work values, as well.

We found that the preoperative GCW was an independent predictor of the postoperative NT-proBNP levels. As a robust marker of congestion, NT-proBNP is strongly associated with the symptomatic status of the patients and also holds an important place in the prediction of long-term outcomes after TAVR [20, 56]. While LV dysfunction is usually considered as the most important trigger of BNP release, LV pressure overload also substantially increases wall stress and therefore, natriuretic peptide secretion even in the presence of maintained or elevated LV contractility [57]. In that sense, NT-proBNP displays a load-dependency similarly to the conventional measures of LV function. Therefore, the preoperatively measured load-dependent functional parameters, such as EF or GLS are also limited in the prediction of the postoperative NT-proBNP levels due to the drastic postprocedural change in the loading conditions. Thus, the afterload-independent myocardial work indices may be superior to them in this regard.

Accordingly, our study suggests that myocardial work indices might be valuable in the assessment of TAVR patients, considering their various associations with the relevant clinical characteristics of this population and their consequential predictive value.

### Limitations

Our study has a few limitations that should be acknowledged for adequate interpretation. This is a single-center study with a limited number of cases—further multicenter expansion of the population would strengthen our findings. Due to the limited patient number, we did not aim to examine the prognostic capabilities of myocardial work in terms of hard endpoints, such as mortality or heart failure events. As the enrollment and follow-up period of the study was during the COVID-19 pandemic, a handful of patients were lost due to missed follow-up visits.

## Conclusions

We found that TAVR improves myocardial work, and accordingly, myocardial work indices could serve as benchmarks for determining the optimal timing of intervention. The inclusion of myocardial work parameters, particularly GWI and GCW, in the evaluation of patients undergoing TAVR might hold significant value in the follow-up. This incorporation of myocardial work parameters into the routine assessment of patients with AS has the potential to enhance our comprehension of cardiac performance in this population by overcoming the limitations associated with other load-dependent echocardiographic parameters. Consequently, clinicians could benefit from these advanced tools to refine the follow-up of LV function after TAVR and to accurately determine the appropriate timing for valve replacement.

## Supplementary Information

Below is the link to the electronic supplementary material.

Supplementary file1 (DOCX 35 KB)

## Data Availability

The datasets presented in this article are not readily available due to patient data privacy regulations. Requests to access the datasets should be directed to the corresponding author and have to be authorized by the local Data Management Committee.
