# Supplementary material for: Non-invasive myocardial work as an independent predictor of postprocedural NT-proBNP in elderly patients undergoing transcatheter aortic valve replacement
Source: GeroScience. 2024 Aug 8;47(3):3311–23. doi: 10.1007/s11357-024-01302-0 (PMC12181568; doi:10.1007/s11357-024-01302-0)
Supplement: Supplementary file 1 — Supplementary file1 (DOCX 35 KB) [file 11357_2024_1302_MOESM1_ESM.docx]

SUPLEMENTARY FILE

**Supplementary Table 1: Comparison of the change in the echocardiographic parameters of those who had increased GWI at the follow-up visit (n=27) and those who did not (n=63)**

|  | LV GWI increased (n=27) | | | LV GWI did not increase (n=63) | | | Preoperative difference |
| --- | --- | --- | --- | --- | --- | --- | --- |
|  | **Preoperative** | **Follow-up visit** | **p-value** | **Preoperative** | **Follow-up visit** | **p-value** | **p-value** |
| LVMi (g/m²) | 133.15±32.69 | 112.06±28.73 | **0.004** | 121.37±38.87 | 101.70±32.86 | **<0.001** | 0.172 |
| LV EDVi (ml/m²) | 69.4±23.3 | 62.0±23.6 | **0.042** | 56.8±20.3 | 53.9±17.0 | 0.160 | **0.012** |
| LV ESVi (ml/m²) | 40.6±21.5 | 31.8±19.3 | **0.005** | 26.2±15.7 | 25.1±13.7 | 0.432 | **0.001** |
| E/A | 1.36±0.60 | 0.92±0.44 | 0.081 | 0.98±0.47 | 0.94±0.45 | 0.619 | **0.018** |
| E/e’ | 19.91±10.50 | 14.84±6.25 | 0.061 | 15.22±5.89 | 14.78±7.19 | 0.621 | **0.031** |
| LAVi (ml/m²) | 45.4±19.7 | 48.4±14.8 | 0.283 | 44.3±16.0 | 46.6±18.5 | 0.183 | 0.791 |
| RAVi (ml/m²) | 30.0±10.8 | 35.5±11.7 | **0.011** | 33.6±15.2 | 37.6±15.1 | **0.012** | 0.490 |
| TAPSE (mm) | 19.5±4.6 | 20.7±5.0 | 0.275 | 22.9±5.3 | 21.6±6.0 | 0.088 | **0.006** |
| PASP (mmHg) | 46.2±13.8 | 40.7±11.3 | 0.200 | 39.8±13.0 | 38.0±14.0 | 0.569 | 0.229 |
| LV EF (%) | 44.7±14.3 | 51.6±11.0 | **0.012** | 56.1±11.1 | 55.4±10.1 | 0.621 | **<0.001** |
| LV GLS (%) | -10.0±4.2 | -14.3±3.6 | **<0.001** | -15.0±3.9 | -15.6±3.8 | 0.083 | **<0.001** |
| LV GWI (mmHg%) | 1247±572 | 1749±632 | **<0.001** | 2199±710 | 1613±605 | **<0.001** | **<0.001** |
| LV GCW (mmHg%) | 1632±683 | 2280±743 | **<0.001** | 2680±714 | 2133±609 | **<0.001** | **<0.001** |
| LV GWW (mmHg%) | 294±188 | 318±152 | 0.480 | 245±143 | 284±140 | 0.083 | 0.182 |
| LV GWE (%) | 83.7±8.2 | 86.3±8.3 | **0.047** | 90.1±6.6 | 86.8±7.8 | **<0.001** | **<0.001** |

Data are presented as mean±SD or median (interquartile range)

Values with a significant difference are presented in bold.

*Abbreviations*: LV = left ventricular, GWI = global myocardial work index, LVMi = left ventricular mass index, EDVi = end-diastolic volume index, ESVi = end-systolic volume index, LAVi = left atrial volume index, RAVi = right atrial volume index, TAPSE = tricuspid annular plane systolic excursion, PASP = pulmonary artery systolic pressure, EF = ejection fraction, GLS = global longitudinal strain, GCW = global constructive work, GWW = global wasted work, GWE = global myocardial work efficiency

**Supplementary Table 2: The correlation analysis for the parameters used in the models of Tables 3-4**

|  | **vs. postoperative NT-proBNP levels** | |
| --- | --- | --- |
|  | **r-value** | **p-value** |
| **Age** | -0.104 | 0.342 |
| **Female sex** | -0.121 | 0.271 |
| **Syncope** | -0.125 | 0.253 |
| **Angina** | 0.108 | 0.323 |
| **Dyspnea** | -0.031 | 0.780 |
| **AS stage** | 0.288 | **0.008** |
| **Diabetes mellitus** | 0.113 | 0.302 |
| **Atrial fibrillation** | 0.427 | **<0.001** |
| **LV GLS (%)** | 0.333 | **0.002** |
| **LV GCW (mmHg%)** | -0.389 | **<0.001** |

Values with a significant correlation are presented in bold.

*Abbreviations*: LV = left ventricular; GLS =global longitudinal strain; GCW = global constructive work

**Supplementary Table 3: Independent predictors of postoperative myocardial work indices: Predictors of postoperative LV GWI**

| Postoperative LV GWI | | | | | |
| --- | --- | --- | --- | --- | --- |
| Clinical characteristics | **β** | **p-value** | **Std. Err.** | **R²** | **Cumulative p-value** |
| Age | 0.086 | 0.347 | 436 | 0.548 | **<0.001** |
| Female sex | **0.450** | **<0.001** |  |  |  |
| AS stage | **-0.280** | **0.002** |  |  |  |
| BSA | 0.014 | 0.890 |  |  |  |
| NYHA stage | -0.127 | 0.143 |  |  |  |
| Diabetes mellitus | **-0.292** | **<0.001** |  |  |  |
| Previous revascularization | 0.048 | 0.561 |  |  |  |
| CIED | **-0.306** | **<0.001** |  |  |  |
| Stroke | **-0.163** | **0.044** |  |  |  |

Values with a significant predictive value are presented in bold.

*Abbreviations*:LV GWI = left ventricular global myocardial work index, AS = aortic stenosis, BSA = body surface area, NYHA = New York Heart Association, CIED = cardiac implantable electronic device

**Supplementary Table 4: Independent predictors of postoperative myocardial work indices: Predictors of postoperative LV GCW**

| Postoperative LV GCW | | | | | |
| --- | --- | --- | --- | --- | --- |
| Clinical characteristics | **β** | **p-value** | **Std. Err.** | **R²** | **Cumulative p-value** |
| Age | 0.080 | 0.414 | 501 | 0.473 | **<0.001** |
| Female sex | **0.414** | **<0.001** |  |  |  |
| AS stage | **-0.276** | **0.004** |  |  |  |
| BSA | 0.021 | 0.844 |  |  |  |
| NYHA stage | -0.155 | 0.097 |  |  |  |
| Diabetes mellitus | **-0.299** | **0.001** |  |  |  |
| Previous revascularization | 0.005 | 0.953 |  |  |  |
| CIED | **-0.242** | **0.008** |  |  |  |
| Stroke | -0.109 | 0.207 |  |  |  |

Values with a significant predictive value are presented in bold.

*Abbreviations*: LV GCW = left ventricular global constructive work, AS = aortic stenosis, BSA = body surface area, NYHA = New York Heart Association, CIED = cardiac implantable electronic device

**Supplementary Table 5: The correlation analysis for the parameters used in the models of Additional Tables 3-4**

|  | **vs. postoperative LV GWI** | | **vs. postoperative LV GCW** | |
| --- | --- | --- | --- | --- |
|  | **r-value** | **p-value** | **r-value** | **p-value** |
| **Age** | 0.148 | 0.168 | 0.164 | 0.125 |
| **Female sex** | 0.434 | **<0.001** | 0.394 | **<0.001** |
| **AS stage** | -0.423 | **<0.001** | -0.416 | **<0.001** |
| **BSA** | -0.279 | **0.009** | -0.256 | **0.016** |
| **NYHA stage** | -0.078 | 0.469 | -0.115 | 0.286 |
| **Diabetes mellitus** | -0.271 | **0.011** | -0.285 | **0.007** |
| **Previous revascularization** | -0.113 | 0.294 | -0.141 | 0.190 |
| **CIED** | -0.357 | **0.001** | -0.283 | **0.007** |
| **Stroke** | -0.170 | 0.114 | -0.114 | 0.291 |

Values with a significant correlation are presented in bold.

*Abbreviations*: LV = left ventricular; GWI =global myocardial work index; GCW = global constructive work; BSA = body surface area; NYHA = New York Heart Association; CIED = cardiac implantable electronic device

**Supplementary Table 6: Comparison of the change in the echocardiographic parameters of those with low flow - low gradient aortic stenosis (n=19) vs. those who do not have low flow - low gradient aortic stenosis (n=71)**

|  | LFLG AS (n=19) | | | Not LFLG AS (n=71) | | | Preoperative difference |
| --- | --- | --- | --- | --- | --- | --- | --- |
|  | **Preoperative** | **Follow-up visit** | **p-value** | **Preoperative** | **Follow-up visit** | **p-value** | **p-value** |
| LVMi (g/m²) | 123.12±30.56 | 111.18±40.45 | 0.105 | 125.39±39.13 | 103.11±29.27 | **<0.001** | 0.816 |
| LV EDVi (ml/m²) | 68.6±29.0 | 62.1±27.5 | 0.066 | 58.4±19.3 | 54.8±16.6 | 0.080 | 0.074 |
| LV ESVi (ml/m²) | 40.8±28.0 | 34.0±23.9 | **0.019** | 27.8±14.4 | 25.3±12.4 | 0.111 | **0.006** |
| E/A | 1.04±0.49 | 1.35±0.58 | 0.340 | 1.07±0.53 | 0.89±0.40 | **0.027** | 0.850 |
| E/e’ | 15.65±9.35 | 14.29±7.87 | 0.685 | 16.71±7.33 | 14.91±6.73 | 0.066 | 0.516 |
| LAVi (ml/m²) | 42.3±20.5 | 45.2±19.4 | 0.391 | 45.2±16.3 | 47.5±17.4 | 0.142 | 0.952 |
| RAVi (ml/m²) | 40.4±25.0 | 45.2±19.4 | **0.012** | 32.9±14.6 | 36.8±14.3 | **0.008** | 0.943 |
| TAPSE (mm) | 19.7±4.9 | 19.8±4.6 | 0.950 | 22.4±5.3 | 21.7±6.0 | 0.317 | 0.064 |
| PASP (mmHg) | 52.6±15.7 | 45.2±19.4 | 0.426 | 39.6±11.9 | 37.5±10.9 | 0.355 | 0.313 |
| LV EF (%) | 46.0±17.3 | 49.9±13.4 | 0.160 | 54.4±11.3 | 55.4±9.3 | 0.479 | **0.012** |
| LV GLS (%) | -11.7±6.1 | -13.3±5.0 | 0.110 | -14.0±4.0 | -15.8±3.2 | **<0.001** | 0.053 |
| LV GWI (mmHg%) | 1454±950 | 1345±601 | 0.470 | 2036±713 | 1737±593 | **<0.001** | **0.004** |
| LV GCW (mmHg%) | 1906±1049 | 1913±679 | 0.965 | 2488±752 | 2248±630 | **0.009** | **0.007** |
| LV GWW (mmHg%) | 252±149 | 336±152 | 0.051 | 262±162 | 283±140 | 0.308 | 0.810 |
| LV GWE (%) | 84.4±9.1 | 82.8±9.8 | 0.337 | 89.2±6.2 | 87.8±6.1 | 0.074 | **0.008** |

Data are presented as mean±SD or median (interquartile range)

Values with a significant difference are presented in bold.

*Abbreviations*; LVMi = left ventricular mass index, LV = left ventricular, EDVi = end-diastolic volume index, ESVi = end-systolic volume index; LAVi = left atrial volume index, RAVi = right atrial volume index, TAPSE = tricuspid annular plane systolic excursion, PASP = pulmonary artery systolic pressure, EF = ejection fraction, GLS = global longitudinal strain, GWI = global myocardial work index, GCW = global constructive work, GWW = global wasted work, GWE = global myocardial work efficiency

**Supplementary Table 7: Inter- and intraobserver variability of the key parameters**

|  | **Intraobserver variability** | **Interobserver variability** |
| --- | --- | --- |
|  | **ICC [95% CI]** | **ICC [95% CI]** |
| **LV GLS** | 0.900 [0.721-0.966] | 0.932 [0.800-0.978] |
| **LV GWI** | 0.900 [0.787-0.953] | 0.886 [0.690-0.961] |
| **LV GCW** | 0.948 [0.851-0.982] | 0.877 [0.678-0.956] |

*Abbreviations*: ICC = intraclass correlation coefficient, LV = left ventricular, GLS = global longitudinal strain, GWI = global myocardial work index, GCW = global constructive work
